# Resource Concentration Modulates the Fate of Dissimilated Nitrogen in a Dual-Pathway Actinobacterium

**DOI:** 10.3389/fmicb.2019.00003

**Published:** 2019-01-22

**Authors:** David C. Vuono, Robert W. Read, James Hemp, Benjamin W. Sullivan, John A. Arnone, Iva Neveux, Robert R. Blank, Evan Loney, David Miceli, Mari-Karoliina H. Winkler, Romy Chakraborty, David A. Stahl, Joseph J. Grzymski

**Affiliations:** ^1^Division of Earth and Ecosystem Sciences, Desert Research Institute, Reno, NV, United States; ^2^Department of Civil and Environmental Engineering, University of Washington, Seattle, WA, United States; ^3^Division of Geological and Planetary Sciences, California Institute of Technology, Pasadena, CA, United States; ^4^Department of Natural Resources and Environmental Science, University of Nevada, Reno, Reno, NV, United States; ^5^Agricultural Research Service, United States Department of Agriculture, Reno, NV, United States; ^6^Earth and Environmental Sciences Area, Lawrence Berkeley National Laboratory, Berkeley, CA, United States

**Keywords:** dissimilatory nitrate reduction, denitrification, ammonification, redox poise, cost minimization, maximum power principle

## Abstract

Respiratory ammonification and denitrification are two evolutionarily unrelated dissimilatory nitrogen (N) processes central to the global N cycle, the activity of which is thought to be controlled by carbon (C) to nitrate (NO_3_^−^) ratio. Here we find that *Intrasporangium calvum* C5, a novel dual-pathway denitrifier/respiratory ammonifier, disproportionately utilizes ammonification rather than denitrification when grown under low C concentrations, even at low C:NO_3_^−^ ratios. This finding is in conflict with the paradigm that high C:NO_3_^−^ ratios promote ammonification and low C:NO_3_^−^ ratios promote denitrification. We find that the protein atomic composition for denitrification modules (NirK) are significantly cost minimized for C and N compared to ammonification modules (NrfA), indicating that limitation for C and N is a major evolutionary selective pressure imprinted in the architecture of these proteins. The evolutionary precedent for these findings suggests ecological importance for microbial activity as evidenced by higher growth rates when *I. calvum* grows predominantly using its ammonification pathway and by assimilating its end-product (ammonium) for growth under ammonium-free conditions. Genomic analysis of *I. calvum* further reveals a versatile ecophysiology to cope with nutrient stress and redox conditions. Metabolite and transcriptional profiles during growth indicate that enzyme modules, NrfAH and NirK, are not constitutively expressed but rather induced by nitrite production via NarG. Mechanistically, our results suggest that pathway selection is driven by intracellular redox potential (redox poise), which may be lowered when resource concentrations are low, thereby decreasing catalytic activity of upstream electron transport steps (i.e., the bc_1_ complex) needed for denitrification enzymes. Our work advances our understanding of the biogeochemical flexibility of N-cycling organisms, pathway evolution, and ecological food-webs.

## Introduction

Globally, respiratory ammonification and denitrification are vital nitrogen (N) dissimilation pathways that either retain reactive N to support net primary productivity or close the N-cycle through the release of gaseous N, respectively (Knowles, 1982). The environmental controls of these two pathways, particularly the ratio of electron-donor to electron-acceptor (e.g., C:NO_3_^−^) (Tiedje et al., 1982), have gained attention (Fazzolari et al., 1998; Schmidt et al., 2011; Kraft et al., 2014; Hardison et al., 2015; van den Berg et al., 2015; Yoon et al., 2015a) due to increased anthropogenic N inputs into the environment (Galloway et al., 2003). Isolating the mechanisms by which pathway selection occurs is challenging and difficult to generalize given the diversity and disparate evolutionary origins of the organisms and the N-reducing modules found in the two pathways. Strong selective pressures from Earth’s shifting biogeochemistry and oxidation-state have driven evolutionary adaptions to microbial electron transport chains (ETC; Moparthi and Hägerhäll, 2011; Dibrova et al., 2013), respiratory chain redox potentials (Schoepp-Cothenet et al., 2009; Bergdoll et al., 2016; Soo et al., 2017), and protein atomic composition (Baudouin-Cornu et al., 2004; Grzymski and Dussaq, 2012). Understanding pathway selection from an evolutionary context may shed light on how these pathways are regulated in contemporary organisms. Here, by identifying the biochemical and evolutionary differences between respiratory ammonification and denitrification, we disentangle the ecological significance and molecular mechanisms of electron transfer through either pathway in a dual pathway organism.

From a biochemical standpoint, the primary difference between respiratory ammonification and denitrification is their respective source of reducing equivalents in the ETC: (1) heme-based cytochrome c nitrite reductase used in respiratory ammonification receive electrons directly from the quinone (Q) pool (Einsle et al., 2002) while (2) copper and *cd*_1_ nitrite reductases used in denitrification receive electrons from a soluble electron carrier (e.g., cytochrome c) via the bc_1_ complex (Kraft et al., 2011). From an evolutionary standpoint, we can place each N-module’s origin to a putative time in Earth history based on the metal co-factors that would have been bioavailable: heme-based cytochromes in an ancient, more reduced, environment compared to the copper-containing nitrite reductases in an oxidizing environment (Godfrey and Falkowski, 2009). The bioenergetic chains of microorganisms also underwent selective pressure to shift from low-potential (LP) to high-potential (HP) quinones in response to Earth’s oxygenation (Bergdoll et al., 2016). Menaquinone (MK) is thought to be the ancestral type of quinone (Nitschke and Dracheva, 1995) and is the most widely used quinone on the phylogenetic tree (Schoepp-Cothenet et al., 2009). Organisms that use ubiquinone (UQ) are thought to have evolved under high O_2_ tensions with α-, β-, γ-proteobacteria as the only bacterial clades to use UQ (Schoepp-Cothenet et al., 2009). Surprisingly, our understanding for the biochemistry of denitrification is based predominantly on HP UQ-based systems (Lalucat et al., 2006), leaving a significant knowledge gap in the physiology and biochemistry of LP MK-based denitrifiers and how they link electron transfer with energy capture under resource limitation (Suharti and de Vries, 2005; Heylen et al., 2012; Decleyre et al., 2016).

It has been postulated that C:NO_3_^−^ ratio modulates the activity of respiratory ammonification versus denitrification (Tiedje et al., 1982). This hypothesis states that high C:NO_3_^−^ ratios (stoichiometric limitation of nitrate relative to C) drive respiratory ammonification while low C:NO_3_^−^ ratios (stoichiometric limitation of C relative to nitrate) drive denitrification. Denitrification theoretically yields more free energy per electron, but respiratory ammonification yields more free energy per nitrate due to reaction stoichiometry: one nitrate is needed to make ammonium while two are needed to make N_2_O/N_2_ (Tiedje et al., 1982; Strohm et al., 2007). Thus, when C:NO_3_^−^ is high, cells select ammonification to maximize the free energy advantage per nitrate. When C:NO_3_^−^ is low, cells select denitrification to maximize the free energy advantage per electron. However, this theory does not account for substrate concentration, which can impose resource limitation on microbial growth. For example, the same C:NO_3_^−^ ratio can be duplicated at two different substrate concentrations. Furthermore, pathway selection occurs at the step of nitrite reduction, not nitrate reduction, and thus low C concentrations would generate low concentrations of nitrite from nitrate. This scenario should then select for the pathway that makes the best use of the free energy advantage per nitrite (i.e., ammonification).

Dual-pathway respiratory nitrite reducers, where respiratory ammonification and denitrification modules are encoded in the same genome, provide a unique system to investigate the molecular mechanisms of C:NO_3_^−^ control on pathway selection. While dual-pathway nitrite reducers are capable of differential energy conservation through pathway bifurcation of nitrite, no mechanistic or metabolic models exist to explain how these dual-pathway organisms partition electron flow and proton translocation between pathways to maximize resource-use efficiency. It is not known if these organisms differentially distribute electron flow through each pathway in response to shifting resource availability or control each pathway independently in response to resource thresholds. In order to resolve the mechanisms of C:NO_3_^−^ control on pathway selection and better understand branched respiratory chains in LP-based N-reducing organisms, we undertook the characterization of *Intrasporangium calvum* C5: A Gram-positive Actinobacterium and dual-pathway nitrite reducer that uses MK as sole pool quinone. We show that over a range of C:NO_3_^−^ ratios (0.1–4), duplicated at two substrate concentrations (mM–μM ranges), batch cultures of *I. calvum* disproportionately utilize its ammonification pathway during low C concentrations (≤ 0.4 mM lactate), when C:NO_3_^−^ ratios are < 1 (an observation contrary to the current paradigm). Using a genome-guided approach coupled to time-series transcriptomics and metabolite profiles, we identified differentially expressed genes in the bacterium’s ETC and central metabolic pathways. Using this information to inform a metabolic reconstruction of the ETC and extensive literature on the biochemistry of the bc_1_ complex, we propose a new mechanism by which these two pathways are regulated at the biochemical level.

## Materials and Methods

### Culture Conditions

#### Sample Collection

*Intrasporangium calvum* was isolated from a groundwater well (GW247) collected on 2/18/2013 at Oak Ridge National Laboratory (Lat: 35.97990, Long: 84.27059) with a groundwater temperature of 15.57°C, conductivity of 521.9 μS/cm, and pH of 7.71. *I. calvum* C5 was isolated and obtained from Dr. R. Chakraborty (Lawrence Berkeley National Laboratory).

#### Media Preparation

All cultures were grown at 30°C and shaken at 250 rpm. Nitrate reducing minimal media was prepared with the following final concentrations: NaCl (0.06 mM), NH_4_Cl (1.4 mM) (for ammonium replete conditions but not used in NH_4_^+^-free conditions), MgCl_2_ (0.2 mM), CaCl_2_ (0.04 mM), KCl (0.1 mM), K_2_HPO_4_ (1.1 mM), NaHCO_3_^−^ (30 mM), cysteine (1 mM) as reducing agent, resazurin as redox indicator, and trace elements and trace vitamin solutions as reported (Yoon et al., 2013; Hillesland et al., 2014). 1 M sterile filtered (0.2 μm) concentrated stocks of 60% w/w sodium DL-lactate solution (Sigma-Aldrich, St. Louis, MO, United States), sodium-nitrate and sodium-nitrite (≥ 99%, Fisher Scientific, Pittsburg, PA, United States) were diluted into media prior to autoclaving to achieve the desired C:NO_3_^−^ ratio. C:NO_3_^−^ ratio was calculated based on (Yoon et al., 2015a) where the number of C atoms (n) in the e-donor is multiplied by the concentration of the e-donor, divided by the number of N atoms in the e-acceptor multiplied by the concentration of the e-acceptor (Supplementary Table S1). See SI Materials and Methods for complete description of Hungate technique prepared media. Mean pH for all culture vessels (time series and end-point; Supplementary Table S2), measured at the end of each experiment, was 7.3 ± 0.05 (*n* = 144).

### Analytical Procedures

#### Growth Curve/Cell Counts/Yield Measurements

Growth curves were measured from scratch-free Balch-tubes grown cultures using an automated optical density reader at OD_600_ nm (Lumenautix LLC, Reno, NV, United States). All experiments began with cells grown in the same media for each respective C:NO_3_^−^ ratio/concentration treatment, which were harvested from late exponential early/stationary phase. This method ensured that the inoculum was in the same nutrient-limited metabolic state when inoculated into fresh media for each respective treatment. Thus, the inoculum was in a nutrient-limited state, as defined by the continuous culture method (Monod, 1950), when each experiment began. End-point cultures were monitored until all replicates reached stationary phase (65–100 h depending on C:NO_3_^−^ treatment) (Supplementary Figure S1). Cell counts were performed by fixing cells in 4% paraformaldehyde (final concentration) for 20 min, filtered onto 0.2 μm pore-sized black polycarbonate filters. A complete description is provided in SI Materials and Methods. Biomass concentrations were measured by filtration and drying as per standard protocol (APHA, 2012). A complete description is provided in SI Materials and Methods.

#### Ion and Gas Chromatography Measurements

A dual channel Dionex ICS-5000+ (Thermo Fisher Scientific) ion chromatograph (IC) was used to measure organic (lactate, acetate, and formate) and inorganic (nitrite and nitrate) anions on an AS11-HC column and cations (ammonium) on a CS-16 column from the bacterial growth media. A complete description is provided in SI Materials and Methods.

#### Nucleic Acid Extraction and Sequencing

DNA for genome sequencing was extracted with phenol-chloroform. The DNA quality and integrity was verified on Agilent 2200 TapeStation and DNA was sequenced on the PacBio platform (Duke Center for Genomic and Computational Biology). For gene expression analysis, *I. calvum* cells were grown in 300 mL batch cultures under 8 mM and 0.8 mM lactate (12 mM and 1.2 mM nitrate, respectively) (*n* = 3). Growth curves were monitored in real-time to collect cells during early-exponential, late-exponential, and stationary phase. Upon sampling, culturing vessels were immediately incubated on ice, aseptically transferred to 50 mL falcon tubes and immediately centrifuged at 15000 G for 5 min at 4°C. Supernatant was immediately poured off and the cell pellets were harvested and stored in TRIzol Reagent (Ambion RNA by Life Technologies) at –80°C until further processing. RNA was extracted with TRIzol Reagent (Ambion RNA by Life Technologies), following the manufacturer’s protocol. The extraction was scaled up to 20 ml of TRIzol and performed in 50 ml conical tubes. RNA was resuspended in water, treated with Baseline-ZERO DNase (Epicenter) and cleaned up with RNeasy MinElute Cleanup Kit (QIAGEN). Total extracted RNA from each growth phase was quantified (RiboGreen RNA quantification), analyzed for RNA integrity number (RIN) (RIN ≥ 0.8 were used for sequencing), treated with RiboZero rRNA removal kit (Illumina, San Diego, CA, United States). Library preparation was performed with the TruSeq Stranded mRNA Library Prep kit (Illumina, San Diego, CA, United States) and sequenced using Illumina NextSeq 500 2 × 150 Mid Output v2 platform, resulting in 10–15 million reads per sample.

### Phylogenetic, Genomic, and Transcriptomic Analysis

Genomic DNA was assembled using Canu (version 1.7.1) with an estimated genome size of five million base pairs (Koren et al., 2017). The resulting single contiguous fragment was aligned to the *I. calvum* 7KIP genome (Acc: NC_014830.1) to compare sequence similarity in Mauve (Darling et al., 2004, 2010). Genome annotation for C5 was performed through the NCBI Prokaryotic Genome pipeline^1^. Additional gene prediction analysis and functional annotation was performed by the DOE Joint Genome Institute (JGI) using the Isolate Genome Gene Calling method (Prodigal V2.6.3 February, 2016) under the submission ID 172966. The complete genome sequence and annotation is available in the NCBI database under the BioProject number PRJNA475609. A complete description of the phylogenetic, pathway analysis, and cost-minimization calculations is provided in SI Material and Methods. For transcriptomic analysis, the resulting raw reads were inspected using FastQC (Andrews, 2010) to determine quality, read length, and ambiguous read percentage. Reads were trimmed based on quality score with a sliding window of five base pairs, quality cutoff of 28, trailing cutoff quality score of 10, as well as adapter contamination removal in Trimmomatic (Bolger et al., 2014). A complete description is provided in SI Materials and Methods. Statistical analyses were conducted in the R environment for statistical computing ^2^. Data that was tested using parametric statistical analysis were first validated for normality by visualizing the data as a histogram and testing via Shapiro–Wilk test for normality.

## Results

### Genomic Analysis of *I. calvum* C5

*Intrasporangium calvum* C5 was isolated from a nitrate contaminated groundwater well (well GW247: > 200 mM nitrate) at the Oak Ridge National Laboratory Field Research Station in Oak Ridge, TN. This species was first isolated from the air in a school dining hall (Kalakoutskii et al., 1967) and has been found in sequence libraries from activated sludge wastewater treatment plants (Vuono et al., 2015) and in other nitrate contaminated groundwater sources (Green et al., 2010). The strain was selected for further analysis due to its nitrate reducing phenotype in minimal media. The genome consists of a 4,025,044-base pair chromosome and encodes for 3,722 predicted genes, 2,665 protein coding genes, 57 RNA genes, and two rRNA operons. We sequenced and analyzed the genome of *I. calvum* C5 to first compare its similarity to the type species *I. calvum* 7KIP. We identified a high degree of sequence similarity to 7KIP based on three homologous sequence regions as locally collinear blocks (SI Results). Genome size of C5 was 4,025,044 base pairs (bp), only 662 bp longer than 7KIP. Genomic analysis of the ETC revealed the typical suite of complexes common to facultative aerobes, including primary dehydrogenases (*nuo* complex, succinate dehydrogenase), alternative *NDH-2* NADH dehydrogenase, cytochrome bc_1_ complex, high-oxygen adapted cytochrome c oxidase (A-family), and low-oxygen adapted cytochrome *bd* oxidase. The bc_1_ complex subunits are also located immediately upstream of cytochrome c oxidase, suggesting that these enzymes are encoded in a single operon creating a supercomplex. Despite *I. calvum*’s propensity for aerobic growth on a number of growth media (Kalakoutskii et al., 1967), employs an unsaturated MK-8 as its sole pool quinone, similar to the type strain (Stackebrandt and Schumann, 2006; del Rio et al., 2010). *I. calvum* also possesses multiple pathways for feeding electrons into the MK-pool, such as formate, malate, hydroxybutyrate, and glycerophosphate dehydrogenases. Once in the MK-pool, there are alternative pathways for MKH_2_ oxidation that can circumvent the bc_1_ complex, such as a membrane-bound respiratory nitrate reductase module (NarG). In addition, to NarG, its dissimilatory N module composition consists of a truncated denitrification pathway (N_2_O is a terminal product) using a copper nitrite reductase NirK and quinol-dependent nitric oxide reductase qNor. *I. calvum* also possesses both catalytic and membrane anchor subunits (NrfA and NrfH, respectively), for a pentaheme cytochrome c module involved in respiratory nitrite ammonification. *I. calvum*’s nitrogen cycling capabilities appear to be unique to the genus as other *Intrasporangium* species, such as *I. chromatireducens* and *I. oryzae* do not possess any known dissimilatory nitrogen cycling genes.

### *I. calvum* Encodes for a Functional NrfAH Complex and Assimilates NH_4_^+^ via Respiratory Nitrite Ammonification

**FIGURE 1:**
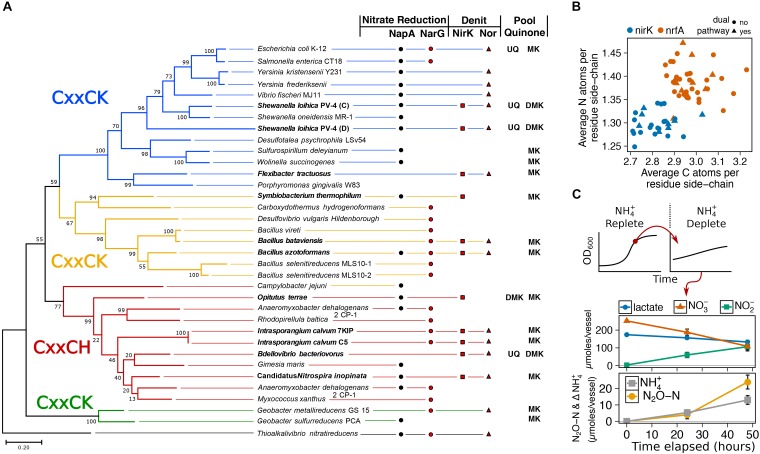
**(A)** Maximum likelihood phylogenetic tree of NrfA amino acid sequences from known respiratory ammonifiers and accompanying N-module composition for each organism. Pool quinone is also noted for dual-pathway nitrite reducers and model species. Colors of the main branches denote the 1st heme motif type: CxxCK and CxxCH. **(B)** Protein atomic composition for N and C normalized to protein length for NirK and NrfA nitrite reductases. **(C)** State-transition from ammonium-replete to ammonium-free for *I. calvum* C5 grown under 8 mM lactate 12 mM nitrate minimal media at 30°C. Metabolite profiles for ammonium-free are shown.

To gain insight into possible function of the NrfAH complex, we aligned the NrfA protein sequences from C5 and 7KIP to a collection of 33 recognized cytochrome c nitrite reductases from published annotated genomes (Supplementary Table S3). This confirmed that NrfA from *I. calvum* is a member of the CxxCH first heme motif group (Figure 1A), which forms one of four clades on the NrfA phylogenetic tree. We then queried the genomes of the taxa in our phylogeny for other annotated N-reducing modules used in nitrate reduction, nitrite reduction, NO-forming nitrite reduction, and primary pool quinone. Among the three major clades of NrfA, at least five additional taxa are noted having dissimilatory N-module inventories containing dual respiratory pathways: *Symbiobacterium thermophilum, Bacillus azotoformans, Bacillus bataviensis*, *Bdellovibrio bacteriovorus*, and *Candidatus* Nitrospira inopinata, (Figure 1A). None of the taxa in our NrfA phylogeny harbored the *cd*_1_ nitrite reductases (NirS). Due to the exclusive NirK representation in dual-pathway membership, we asked whether there might be differences in protein atomic composition between NirK and NrfA, given the disparate evolutionary origins of these modules (Klotz and Stein, 2008). We collected 20 additional publicly available NirK protein sequences from non-dual-pathway denitrifiers (Supplementary Table S3) and calculated the protein C and N composition for our NirK/NrfA collection as atoms per residue side-chain (Figure 1B). These results showed a significant depletion in C and N atoms per residue side-chain (ARSC) for NirK compared to NrfA (C and N: *p* < 0.001; *t*-test), indicating that resource constraints are imprinted on the evolution of these proteins.

The potential routes for N-assimilation were screened using aerobic minimal media with defined C-source/e-donor and e-acceptor, and with nitrate or ammonium as assimilatory N-sources. Based on genomic information, the bacterium possesses no known assimilatory nitrate reductase, but encodes for an ammonium transporter (Intca_RS11655) and GS/GOGAT pathways (Intca_RS13810; Intca_RS11930; Intca_RS08335, 08340), suggesting that ammonium is its sole assimilatory N source. We observed no aerobic growth with 8mM lactate, O_2_, and nitrate as N-source. However, when grown on 8 mM lactate, O_2_, and 1.5 mM ammonium as N-source, *I. calvum* displayed a typical growth curve with a specific growth rate of 0.4 ± 0.02 μ (1.7 ± 0.1 doublings/hour) (Supplementary Figure S2). The functionality of *I. calvum*’s Nrf complex was then tested by growing the bacterium under reducing conditions (8 mM lactate, 12 mM nitrate, ammonium-replete). We then performed a state-transition where biomass from late-exponential growth phase was collected and anaerobically inoculated into ammonia-free media (Figure 1C). Despite no detectable amounts of ammonium produced in the media over time, cell counts increased by 29% over the inoculum in the ammonium-free media (a factor of 3.5×, or 1.8 generations with a net total of 5.4 × 10^5^ ± 8.9 × 10^4^ cells/mL) over a 48 h incubation, indicating consumption of ammonium produced by NrfA. Net ammonium production was 13 ± 2.7 μmoles with the remainder of dissimilated N being used by the denitrification pathway (24 ± 4.2 μmoles N_2_O-N), resulting in a recovery of 97.4% dissimilated N. These results confirmed that *I. calvum* C5 has a functional Nrf complex and also consumes the product (ammonium) of respiratory nitrite ammonification.

### Respiratory Nitrite Ammonification Exceeds Denitrification Under Low C Concentrations

**FIGURE 2:**
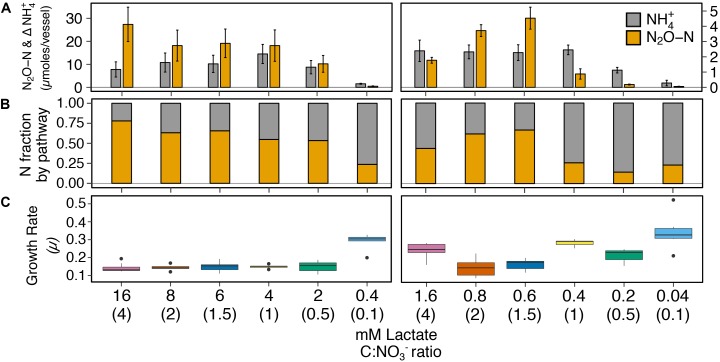
The effects of high resource (left; range of lactate concentrations with 12 mM NO_3_**^−^**) and low resource (right; range of lactate concentrations with 1.2 mM NO_3_**^−^**) concentrations with the same C:NO_3_**^−^** ratio on pathway selection in *I. calvum* C5. **(A)** Production of N_2_O-N and net change of NH_4_^+^ over a 100-h incubation period at 30°C. Each bar represents the average of 8–10 replicates per treatment (Supplementary Table S2). **(B)** Fraction of dissimilated N by pathway. **(C)** Growth rates for each corresponding treatment. The *x*-axis label defines lactate concentration and C:NO_3_**^−^** ratio in parentheses.

We investigated C:NO_3_^−^ control on respiratory ammonification versus denitrification on cultures of *I. calvum* C5 over a high resource C:NO_3_^−^ range (16–0.4 mM lactate, 12 mM nitrate; ratio 4–0.1) and low resource C:NO_3_^−^ range (1.6–0.04 mM lactate, 1.2 mM nitrate; ratio 4–0.1). Under all the treatments tested, results showed products of both respiratory pathways, differing only in the relative fraction of N_2_O versus ammonium production across treatments (Figure 2). At high resource concentrations, respiratory ammonification did not prevail at high C:NO_3_^−^ ratios (Figures 2A,B, left panels; Supplementary Table S4). Instead, significantly greater amounts of N_2_O were produced over ammonium, though nitrite was still the major extracellular end-product of nitrate respiration. Despite the predominance of N_2_O production under the high resource concentrations, ammonium production exceeded N_2_O production only at the lowest C:NO_3_^−^ ratio (0.4 mM lactate, ratio = 0.1; Figure 2) and accounted for 76.2 ± 0.1% of dissimilated N.

Results from the low resource dataset provided weak support for the strict stoichiometry hypothesis that C:NO_3_^−^ controls pathway selection. Ammonia exceeded N_2_O production only under one high C:NO_3_^−^ ratio treatment (ratio = 4; 1.6 mM lactate; Figures 2A,B, right panels). However, at ratios ≤1 (≤0.4 mM lactate), significantly more ammonium than N_2_O was produced. On average, respiratory ammonification accounted for 78.1 ± 8.9% of dissimilated N for lactate concentrations ≤0.4 mM (Supplementary Table S4). When these results are taken in context with cell physiology, we observed a significant and positive relationship between specific growth rate (μ) and the fraction of N dissimilated by respiratory ammonification (*R^2^* = 0.5; *p* < 0.001; Figure 2C, Supplementary Figure S3, and Supplementary Table S5).

### Resource Concentration Influences the Metabolite Profiles of Ammonium and N_2_O Production

**FIGURE 3:**
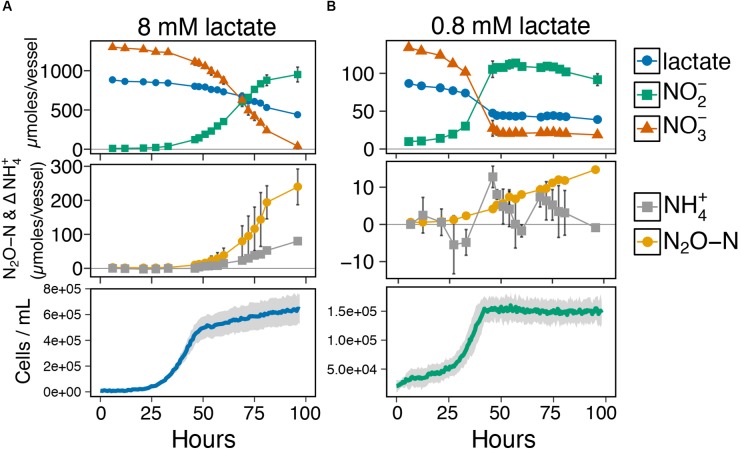
**(A)** Time-series metabolite profiles for lactate, nitrate, and nitrite (top pane), production of dissimilated end-products as N_2_O-N and net change in NH_4_^+^ ammonium production (middle pane), and corresponding growth curve of *I. calvum* cells grown under 8 mM lactate 12 mM nitrate (C:NO_3_^−^ ratio = 2; bottom pane). **(B)** Time-series metabolite profiles for lactate, nitrate, and nitrite (top pane), production of dissimilated end-products as N_2_O-N and net change in NH_4_^+^ ammonium production (middle pane), and corresponding growth curve of *I. calvum* cells grown under 0.8 mM lactate 1.2 mM nitrate (C:NO_3_^−^ ratio = 2; bottom pane).

Given the co-occurrence of end products from both pathways during the end-point experiments (Figure 2), we next investigated the timing of ammonium and N_2_O production relative to metabolite profiles for lactate, nitrate/nitrite, and growth phase at two resource concentrations with the same ratio (8 mM and 0.8 mM lactate, ratio = 2; Figures 3A,B and Supplementary Figure S4). Despite ample e-donor and e-acceptor available for growth, the growth rate at the high resource cultures slowed significantly at ∼50 h (Figure 3A). Metabolite profiles showed that ammonium and N_2_O production began simultaneously, as soon as nitrite was produced from nitrate reduction. The low resource cultures entered stationary phase at ∼40 h (Figure 3B) after nitrate had been fully utilized. No further cell growth was observed after stationary phase was reached. These results show that cell growth occurred primarily on the reduction of nitrate, while nitrite reduction to ammonium and N_2_O occurred during stationary phase. These results indicate that growth is not coupled to nitrite reduction in *I. calvum*. The metabolite profiles for ammonium and N_2_O at low resources (Figure 3B) did not mirror those observed at high resources (Figure 3A). The rate of N_2_O production significantly decreased and ammonium production oscillated rather than steadily increase through time. These differences in metabolite profiles, further demonstrate that concentration influences the activities of pathway bifurcation in *I. calvum*. Repeated time series experiments that were extended up to 300 h showed that nitrite was slowly depleted, but did not get fully consumed under high and low resource concentrations (Supplementary Figure S4). When cultures were given nitrite, instead of nitrate as a terminal electron acceptor (8 mM lactate, 12 mM nitrite; ratio = 2), we observed no immediate growth (as was observed with nitrate) but measured more N_2_O than ammonium production (33.4 ± 4.8 μmoles N_2_O-N and 8.0 ± 2.5 μmoles NH_4_^+^, respectively) (Supplementary Figure S5). These results demonstrate that respiratory ammonification does not exceed denitrification under this particular resource concentration when nitrite is supplied as the sole acceptor with *I. calvum*.

### Nitrite-Reducing Modules Are Up-Regulated During Late Exponential-and Stationary-Phase Growth

**FIGURE 4:**
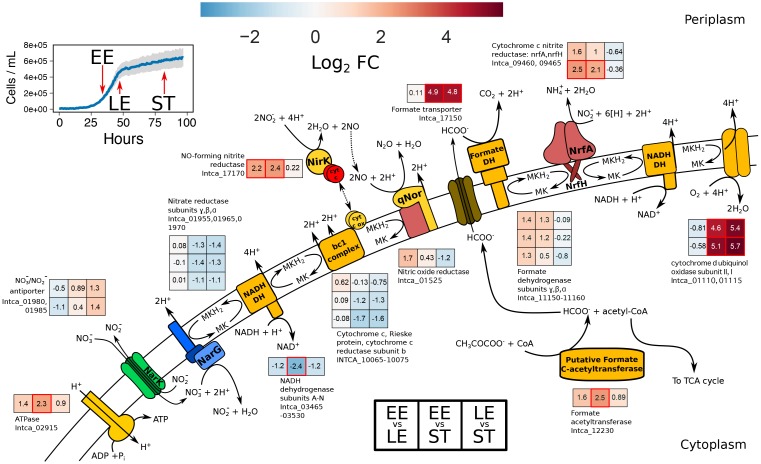
Metabolic reconstruction of the ETC from *I. calvum* with transcriptional changes for genes participating in dual-pathway dissimilatory nitrite reduction. Sampling points for transcriptomic profiling are shown on the inset growth curve with red arrows for each respective growth phases. Log_2_ fold changes in transcript abundance are shown for late exponential relative to early exponential growth phase (EE vs. LE), stationary phase relative to early exponential growth phase (EE vs. ST), and stationary phase relative to late exponential growth phase (LE vs. ST). Locus IDs for each gene product correspond to heat map subplots in the order shown (left-to-right for each growth phase and top-to-bottom for each locus ID specified). Higher transcript abundance is represented in red, lower transcript abundance in blue, and no change in transcript abundance in white. Significant changes in transcript abundance (*p* < 0.01) are marked as a red box. Value of log_2_ fold change is specified within each subplot. The log_2_ fold changes of 14 NADH dehydrogenase subunits (Intca_03465-03530) were averaged as transcriptional changes were all shifted in the same direction.

In order to gain insight into mechanisms of gene regulation and transcriptional organization of *I. calvum*, we conducted RNA-Seq in parallel with the high resource time-series metabolite profile (Figure 3A). This approach enabled us to compare genome-wide differential expression based on log_2_ fold change (lfc) of RNA extracted from three growth phases: early exponential (EE), late exponential (LE), and stationary (ST; Figure 4 and Supplementary Figure S6). Within the central metabolic pathway beginning with the conversion of lactate to pyruvate, we observed a moderate decrease in transcript abundance of L-lactate dehydrogenase (LDH) (Intca_16740) between EE-LE and -ST (lfc = −1.6 ± 0.7; −1.9 ± 0.7), respectively. Lactate utilization protein C (LUP) (Intca_04080), an enzyme involved in lactate degradation, also showed a moderate and significant decrease in transcript abundance between EE-LE and -ST (lfc = −1.6 ± 0.6; −2.4 ± 0.6, *p* = 0.002), respectively. Some acetate was observed in the media (100–200 μM range), indicating minor incomplete lactate oxidation, however, *I. calvum* predominately oxidizes lactate completely to CO_2_. *I. calvum* encodes for two parallel metabolic pathways for pyruvate conversion to acetyl-CoA: pyruvate dehydrogenase (PDH) (Intca_01255) and pyruvate ferredoxin oxidoreductase (PFOR) (Intca_15510). For PDH, there was a significant and moderate increase in transcript abundance between EE-LE and -ST (lfc = 2.1 ± 0.6, *p* = 0.002; 1.5 ± 0.6), respectively. For PFOR, there was a minor decrease in transcript abundance between EE-LE (lfc = −0.43 ± 0.5), and then a moderate increase in transcript abundance between EE-ST (1.1 ± 0.5). Citrate synthase (Intca_04135), the enzyme catalyzing the conversion of acetyl-CoA to citrate and the first step of the tricarboxylic acid (TCA) cycle, showed a highly significant increase in transcript abundance between EE-LE and -ST (lfc = 4.3 ± 0.5, *p* < 0.001; 6.9 ± 0.5, *p* < 0.001).

Within the ETC, there was moderate and significant decrease in transcript abundance for all subunits from the primary dehydrogenase (*nuo* complex; Intca_03465–03539) between EE-LE and -ST (lfc = −1.2 ± 0.3; –2.4 ± 0.6, *p* < 0.001), respectively. Nitrate reductase subunits showed no change in transcript abundance between EE-LE (lfc = 0.01 ± 0.07) and moderately decreased in abundance by ST (lfc = −1.2 ± 0.1), which was corroborated by the depletion of nitrate during stationary phase. There was a significant increase in transcript abundance of *nirK* (Intca_17170) (lfc = 2.2 ± 0.6, *p* = 0.003; 2.4 ± 0.6, *p* < 0.001) and quinol dehydrogenase/membrane anchor subunit *nrfH* (Intca_09465) (lfc = 2.5 ± 0.6, *p* = 0.001; 2.1 ± 0.6, *p* = 0.003) by EE-LT and EE-ST, respectively, which coincided with nitrite production (Figure 3A). The catalytic subunit of the cytochrome c nitrite reductase complex (*nrfA*) (Intca_09460) also increased moderately in transcript abundance by EE-LT and EE-ST (lfc = 1.6 ± 0.6; 1.0 ± 0.6), respectively. Contrary to the transcript abundance patterns of *nirK* and *nrfAH*, nitric oxide reductase (qNor; Intca_01525) transcripts moderately increased between EE-LT (lfc = 1.6 ± 0.6) but decreased in the successive time periods (lfc = 0.43 ± 0.6 between EE-ST; lfc = −1.2 ± 0.6 between LE-ST).

There was a significant increase in transcript abundance of formate transporter *focA* (Intca_17150) between EE-ST, as well as LE-ST (lfc = 4.9 ± 0.7, *p* = 0.002; 4.8 ± 0.7, *p* = 0.002; respectively). We verified the production of formate in our ion chromatography measurements in the range of 100–200 μM following late exponential growth. We also observed a moderate increase in transcript abundance of formate dehydrogenase (FDH) subunits (Intca_11150–11160). These results implicate the activity of formate oxidation, which would contribute to a proton motive force in the periplasm via a Q-loop mechanism and the reduction of MK for electron transfer to nitrite via cytochrome c nitrite reductase. Considering that formate was not provided in our media recipe, an alternative pathway for formate production must exist in *I. calvum*. We also observed acetate production in similar concentrations as formate (100–200 μM). In *E. coli*, formate is produced anaerobically from the action of pyruvate formate lyase (PFL). We identified a putative PFL based on genome annotation (Intca_12230), where transcript abundance also significantly increased by ST. PFL is also highly sensitive to oxygen (Abbe et al., 1982), which was also in agreement with a significant increase in transcript abundance between EE-ST and LE-ST of cytochrome *bd* oxidase (Intca_01110 and Intca_01115), which is thought to protect anaerobic enzymes against oxidative stress (Das et al., 2005).

## Discussion

In this study, we tested the effects of C:NO_3_^−^ ratio and substrate concentration on pathway selection between respiratory ammonification and denitrification in the dual-pathway organism *I. calvum* C5. We challenge the paradigm that C:NO_3_^−^ ratio controls pathway selection based on a simple principle: ratios do not account for substrate concentrations, which can impose resource limitation for C or NO_3_^−^. We hypothesized that limitation for C or NO_3_^−^ should better predict pathway selection in a dual-pathway denitrifier/respiratory ammonifier. To test this hypothesis, we systematically measured the response of batch cultures of *I. calvum* (inoculated under a nutrient-limited metabolic state) to high and low resource loadings under the same range of C:NO_3_^−^ ratios to better resolve mechanisms of pathway selection. We demonstrated that resource concentration, not C:NO_3_^−^ ratio, influences pathway selection in *I. calvum*. We found stronger support for the preference of respiratory ammonification under low C concentrations (at low C:NO_3_^−^ ratios) in *I. calvum*, which grew at significantly higher growth rates when using its ammonification pathway (Figure 2). These results suggest that the NrfA complex, which receives electrons directly from the MK-pool, is optimized to take advantage of the higher Δ*G* per nitrite to generate a proton motive force. The enrichment of C and N ARSC in publicly available NrfA over NirK protein sequences (Figure 1B) provides further support and evolutionary precedence to ammonification preference over denitrification under low resource concentrations where nutrient limitation occurs. This is because the end-product of ammonification can be used as an assimilatory N-source (Figure 1C), indicating that there is no evolutionary constraint to cost minimize N in the Nrf complex. These data, together with metabolic reconstructions (Figure 4) from metabolite and transcriptional profiles suggests that C:NO_3_^−^ ratio alone is insufficient to explain pathway selection.

The theoretical basis for pathway selection can be explained by the law of the minimum (LM; Liebig, 1840) and the maximum power principle (MPP), which state that growth is limited by the least abundant resource and that biological systems are designed to maximize power in order to effectively allocate energy to reproduction and survival (Lotka, 1922; DeLong, 2008), respectively. Here, it appears these two theories are working together: when a growth limiting resource is in short supply, the cell utilizes the respiratory pathway that is optimized to maximize power. Power, in this case, is realized as higher growth rates from the cultures exhibiting disproportionately higher ammonium production than N_2_O production (Figure 2: high resources: C:NO_3_^−^ ratio = 0.1; low resources: C:NO_3_^−^ ratios = 4, 1, 0.5, 0.1). This framework is further bolstered by the observation that growth yields of pure denitrifiers are significantly lower than expected compared to ammonifiers (Strohm et al., 2007). This suggests that despite the lower free energy yield, ammonification conserves more energy during catabolism, through the generation of a proton motive force, to build biomass during anabolism. More specifically, the bacterium must generate a greater proton motive force in order to maximize power when starved for a growth limiting resource. This may help to further explain how respiratory ammonification, which is overall energetically less favorable than denitrification (lactate with nitrite: Δ*G* = −781 versus Δ*G* = −1248, respectively), can have higher growth yields (Strohm et al., 2007) and growth rates (Figure 2 and Supplementary Figure S1) under C- and N-limitation due to the higher energy yield on a per-nitrite basis (denitrification: −217 KJ per mole nitrite; respiratory ammonification: −399 KJ per mole nitrite). For comparison, a total of 8 H^+^ are translocated during denitrification by *I. calvum* (not including nitrate reduction since both pathways share this step; Figure 4): NADH dehydrogenase translocates 4 H^+^ per MKH_2_ oxidized and the bc_1_ complex translocates an additional 4 H^+^ per MKH_2_ oxidized. However, 2 H^+^ must be consumed in the periplasm to reduce nitrite to NO (Zumft, 1997). qNor has a net zero H^+^ release (consumes 2 H^+^ to make N_2_O but releases 2 H^+^) without MKH_2_ regeneration (Hemp and Gennis, 2008). Thus, a net total of 6 H^+^ are translocated per nitrite reduced in denitrification with added biosynthetic costs of making the bc_1_ complex and qNor. In respiratory ammonification, MK/MKH_2_ redox pair is cycled between NADH dehydrogenase and formate dehydrogenase. 6 electrons and 8 H^+^ are needed to reduce nitrite to ammonium, thus 3 MKH_2_ are needed (Einsle et al., 2002). If MKH_2_ is received from NADH dehydrogenase, 12 H^+^ are translocated plus 2 H^+^ from FDH. As each MKH_2_ is oxidized at the binding site of NrfH, 2 H^+^ are liberated (Einsle et al., 2002), resulting in a net total of 12 H^+^ translocated per nitrite reduced for respiratory ammonification. This implies that the cell might deplete its NADH pool more rapidly on a per nitrite basis. However, if more protons are pumped in the early stages of growth, the cell would be allocating the ATP generated for anabolism, as evidenced by higher growth rates in the cultures exhibiting higher amounts of respiratory ammonification (Figure 2), which is supported by the MPP.

Under our high resource conditions (Figure 2; left panels), at C:NO_3_^−^ ratios ≥ 1, we observed that denitrification prevailed and these cultures had lower growth rates than the predominantly ammonium producing cultures. These high resource circumstances resulted in the production of toxic intermediates (i.e., NO_2_^−^ and possibly NO, albeit at undetectable levels), which may explain why these cultures had lower growth rates (Figure 2; left panels) and slowed growth after 50 h in our high resource metabolite profile (Figure 3A). Rowley et al. (2012) reported that at least 20% of nitrate consumed was released as N_2_O from the competition between nitrite and nitrate in the active-site of NarG, when nitrite was present in mM amounts. Under our excess C concentrations, some of the N_2_O production may have been generated via this non-specific activity by NarG. The intracellular production of NO from NO_2_^−^ by NarG are likely inhibitory to cell growth, which may explain why our growth curves (Figure 3A) slowed significantly before nitrate had been fully utilized (as compared to the low resource metabolite profile, Figure 3B). Furthermore, C and N concentrations were rather high under these conditions and the cells were likely experiencing toxicity from NO and NO_2_^−^. Because of these higher resource concentrations, the metabolic outcomes would be beyond the scope of the LM and MPP. Nonetheless, these results clearly demonstrate that end-product formation from the two resource concentrations tested, with the same C:NO_3_^−^ ratio, were not identical. This observation calls into question the generality of the C:NO_3_^−^ control hypothesis on pathway selection.

We selected a single treatment (8 mM lactate, 12 mM nitrate; C:NO_3_^−^ ratio = 2), in which we observed both denitrification and respiratory ammonification occurring simultaneously, for RNA-Seq in order to gain insight into the transcriptional organization of actively growing *I. calvum* cells (Figure 4). Strangely, we saw a decrease in transcript abundance encoding for two enzymes known to convert lactate to pyruvate, LDH and LUP. While normalized read counts (Supplementary Figure S6) were generally consistent across growth phases, indicative of constitutive expression, further research investigating the mode of anaerobic lactate oxidation in *I. calvum* would illuminate how reducing equivalents are fed into its central metabolic pathway. For example, *S. loihica* PV-4 is known to use lactate for both denitrification and respiratory ammonification, but only uses acetate for denitrification (Yoon et al., 2013). Nonetheless, our transcriptomic data suggests that pyruvate plays a central role in providing reducing equivalents to the TCA cycle as Acetyl-CoA, as evidenced by significant upregulation in the genes encoding for pyruvate dehydrogenase and citrate synthase, as well as apparent “leaking” via incomplete lactate oxidation through the release of acetate and formate. Such leaking may be produced by a putative PFL, adding to the diversity of C utilization pathways feeding the ETC, and thereby driving pathway selection for nitrite reduction. Our transcriptomic results, coupled with a parallel metabolite profile (Figure 3A), also suggest that the dual-pathway is induced by the presence of nitrite, and is not constitutively expressed like nitrate reductase, *narG*. Furthermore, it appears that the significant increase in transcript abundance for the gene encoding the *bd* oxidase helps to protect the anaerobic-dependent biochemical machinery against oxidative stress, thereby scavenging any residual oxygen during anaerobic growth.

Our metabolite profiles for N oxyanion respiration and N_2_O versus ammonium production show conflicting patterns relative to previous studies (Figures 3A,B). Yoon et al. (2015b) reported complete reduction of nitrate, production of nitrite, and then rapid consumption of nitrite, with N_2_O as the main end-product, by *S. loihica* PV-4 (5 mM lactate, 1 mM nitrate; ratio = 0.6). When Yoon et al. (2015b) replaced nitrate with nitrite as the dominant electron acceptor (5 mM lactate, 1 mM nitrite, ratio = 0.6), ammonification prevailed. Other research has shown the same response to nitrite replacement and ammonification dominance using non-fermentable C-sources (i.e., acetate) in chemostat enrichments of *Geobacter lovleyi* (van den Berg et al., 2017). In our work, nitrite was never fully depleted (Figures 3A,B and Supplementary Figure S4) and when nitrite was given as the only electron acceptor, the bacterium predominantly used denitrification but without concurrent growth (Supplementary Figure S5). Similar to our work, Kraft et al. (2014) also reported denitrification dominance when nitrite was supplied as the terminal acceptor. In our case, it is possible that the high nitrite concentrations (12 mM) used in the experiment selected for denitrification as a relief valve to combat nitrite toxicity. Nonetheless, more research is needed to distinguish the effects of nitrite availability on pathway selection in organisms that contain different pool quinones.

The generality of our results on *I. calvum* to microbial communities found in nature requires further investigation. The N-cycle is composed of many enzyme modules, many with the same function but different structural and evolutionary origin, such as NirK and NirS (Jones et al., 2008), and these differences may have functional implications. For example, *S. loihica* PV-4 (a Gram-negative dual-pathway γ-proteobacterium) and *I. calvum* use different types of nitrate reducing modules. *S. loihica* PV-4 utilizes NapA whereas *I. calvum* utilizes NarG (Figure 1A). The latter translocates 2 H^+^ per nitrate reduced while NapA consumes 2 H^+^ in the periplasm (Kraft et al., 2011). Both reductases would generate a proton motive force via NADH dehydrogenase H^+^ translocation, but the NapA module would result in a net loss of 2 H^+^, which may impact the selection of downstream respiratory modules. For example, given the lower-than-expected observed growth yields of denitrifiers compared to respiratory ammonifiers (Strohm et al., 2007), if a NapA module is used, it would make sense for respiratory ammonification to be selected under high C:NO_3_^−^ ratios because the cell would need to compensate for less energy conservation during nitrate reduction. Nitric oxide reductase composition may also impact pathway selection. For example, qNor does not translocate H^+^, while sNor, eNor, and gNor are predicted to conserve energy through H^+^ translocation (Hemp and Gennis, 2008). Thus, the modularity of dissimilatory N reduction processes, and whether those modules conserve energy or not, may impose certain constraints on pathway selection in different organisms and should be further investigated.

A detailed look into the biochemistry of ETC complexes may also help shed light on the molecular mechanisms modulating pathway selection. For example, Yoon et al. (2015a) demonstrated that elevated pH selects for ammonification in *S. loihica* PV-4. This phenotypic response is due to a decrease in the midpoint potential of the Rieske protein at higher pH (Trumpower, 1990; Link et al., 1992; Ugulava and Crofts, 1998; Zu et al., 2003). Thus, any hindrance of electron flow through the bc_1_ complex would ultimately reduce the activity of downstream processes and promote alternative respiratory pathways. Nitrogen and C limitation have also been shown to influence flux distributions in redox sensitive proteins, including those found in electron transport (Ansong et al., 2014). A drop in the intracellular redox potential (redox poise) of the cell due to resource concentration may also decrease the midpoint potential of the Rieske protein and reduce the activity of any downstream electron exit modules, such as NirK (Denke et al., 1998; Snyder et al., 1999; Hunte et al., 2008). Thus, based on fundamental principles of protein redox chemistry and thermodynamics, it appears that denitrification versus ammonification are likely not modulated by an arbitrary ratio of C:NO_3_^−^, but rather by thermodynamic constraints of the Q-cycle (Schoepp-Cothenet et al., 2009; Bergdoll et al., 2016). The phenotypic response of higher rates of denitrification over ammonification at high C:NO_3_^−^ ratios in other published studies (van den Berg et al., 2015; Yoon et al., 2015a) may also be due to enrichment bias for organisms that utilize quinones with higher midpoint potentials in their bioenergetic chains (Figure 1). Bergdoll et al. (2016) suggested that comparisons of Rieske/cyt*b* complexes from organisms with high- and low-potential quinones may help to reconcile the thermodynamic properties of Q-cycle function. However, most of our understanding of denitrification bioenergetics is based on evolutionarily recent UQ-based HP bioenergetic chains from Gram-negative α-, β-, γ-proteobacteria. Because *I. calvum* uses a MK-based LP bioenergetic chain it may be possible that the differences in pathway selection across treatments are unique to LP chains.

Piecing together the evolutionary history of the N-cycle using isotopic signatures for geochemically available N module cofactors (i.e., Ni, Fe, and Mo) coupled to molecular evolutionary analysis has revealed respiratory ammonification was likely a major component of the Archean N-cycle (Klotz and Stein, 2008). Abiotic nitrite formation and depletion of ammonia through photodissociation (Falkowski, 1997) would have created selective pressures for a dissimilatory N pathway that also produced assimilatory N. We demonstrate that NrfA proteins are significantly enriched in N compared to NirK [i.e., no evolutionary constraints to cost minimize N in the *nrfA* gene product (Grzymski and Dussaq, 2012)] (Figure 1B) and that ammonium production (without accumulation in the medium) supports growth in *I. calvum* (Figure 1C). The Nrf module is also relatively simplistic in that it receives electrons directly from the quinol pool and not the bc_1_ complex used in denitrification. The early exit of electrons from the ETC (i.e., before reaching the bc_1_ complex) suggests that Nrf may have originated prior to the bc_1_ complex. Furthermore, the quinol oxidation site (Q_o_) of cytochrome *b* contains a PDWY motif, indicative of an ancestral LP respiratory chain found in many Gram-positive organisms (Kao and Hunte, 2014). However, there is still debate regarding the presence of a cytochrome *bc*_1_ complex in the last universal common ancestor (Dibrova et al., 2013; Kao and Hunte, 2014). Lastly, the Nrf module is wired to operate via a Q-loop with formate dehydrogenase whose Mo-cofactors would have also been bioavailable during the Archean, further supporting an early evolution.

In summary, we employ a new predictive framework that accounts for the biochemistry and evolutionary history of N modules, ETC complexes, and pool quinones to suggest the mechanisms by which these two pathways are regulated at the molecular level. With this understanding, it may be possible to extend our framework to environmental microbial populations and accelerate model development across different ecosystem scales (i.e., cross-scale systems biology).

## Author Contributions

DV wrote the manuscript. DV, RR, M-KW, RC, DS, and JG contributed to the conception and designed the study. DV, BS, IN, RB, EL, and DM performed the lab work. DV, RR, JH, BS, JA, RB, M-KW, DS, and JG analyzed the data and interpreted the results. All authors contributed to manuscript revision, read, and approved the submitted version.

## Conflict of Interest Statement

The authors declare that the research was conducted in the absence of any commercial or financial relationships that could be construed as a potential conflict of interest.

## Footnotes

1 www.ncbi.nlm.nih.gov/genome/annotation_prok/

2 r-project.org
