# Supplementary material for: Resource Concentration Modulates the Fate of Dissimilated Nitrogen in a Dual-Pathway Actinobacterium
Source: Front Microbiol. 2019 Jan 22;10:3. doi: 10.3389/fmicb.2019.00003 (PMC6349771; doi:10.3389/fmicb.2019.00003)
Supplement: Supplementary file 1 [file Data_Sheet_1.pdf]

Supplementary Material

Resource concentration modulates the fate of dissimilated nitrogen in a dual-pathway Actinobacterium

David C. Vuono, Robert W. Read, James Hemp, Benjamin W. Sullivan, John A. Arnone III, Iva Neveux, Robert R. Blank, Evan Loney, David Miceli, Mari Winkler, Romy Chakraborty, David A. Stahl, Joseph J. Grzymski*

*** Correspondence:** Joseph J. Grzymski: Joe.Grzymski@dri.edu

This PDF file includes:

Supplementary Materials and Methods
Figs. S1 to S6
Tables S1 to S5
SI reference citations

SI Materials and Methods

Media preparation: Media preparation was conducted in a 2L Widdel Flask. After autoclaving, the media was immediately put under an anoxic headspace (N2/CO2 80:20 mix) and sterile filtered (0.2µm) trace elements, trace vitamins, and reducing agent were added. The media was cooled under an anoxic headspace and buffered with bicarbonate to maintain a pH of 7.2. Hungate technique was used to dispense media into culture tubes (20 mL) and serum vials (100 mL) pre-flushed with a sterile stream of ultra-high purity (UHP) N_2 and sealed with blue 1" butyl rubber stoppers. End-point cultures were grown in Balch tubes (18x150-mm glass tube) sealed with butyl rubber stoppers. Cultures for time-course sampling were grown in 160ml serum vials. All end-point experiments were terminated after 100 hours unless otherwise noted.

Growth Curve/Cell counts/Yield Measurements: Growth curves were measured from scratch-free Balch-tubes grown cultures using an automated optical density reader at OD_{600} nm (Lumenautix LLC, Reno, NV). End-point cultures were monitored until all replicates reached stationary phase (65-100 hours depending on C:NO_3^- treatment) (Figure S7).

Cell counts were performed by fixing cells in 4% paraformaldehyde (final concentration) for 20 minutes, filtered onto 0.2µm pore-sized black polycarbonate filters, and washed three times with phosphate buffered saline (PBS, pH 7.2). Filtered cells captured on the black polycarbonate filters

were stained with SYBR® Gold nucleic acid stain (10-minute incubation) (ThermoFisher Scientific) and counted manually with a fluorescence microscope (Olympus BX60, Tokyo, Japan). We collected cells from during lag phase, exponential phase, and stationary phase in order to create a standard curve of cell counts versus optical density (OD_{600}). We fit a linear model to cell count versus OD_{600} (R^2=0.99) and used the resulting linear equation for cell count enumeration for growth curves during our various treatment conditions.

Biomass concentrations were measured by filtration and drying as per standard protocol (APHA, 2012) for 8mM lactate/12mM nitrate and 0.8mM lactate/1.2mM nitrate treatments and conducted in parallel with growth curve/cell counts as described above. Analysis from triplicate cultures yielded (0.064 ± 0.003) and (0.016 ± 0.001) mg of biomass (dry weight) ml^{-1} for 8mM and 0.8 mM lactate cultures, respectively. Cell counts from stationary phase cultures were (1.5 ± 0.05) x 10^7 and (1.16 ± 0.09) x 10^6 for 8mM and 0.8 mM lactate cultures, respectively. From these values the dry weight of a single *I. calvum* cell was estimated to be 1.09 x 10^{-10} g. Growth yield (*Y*) (Table S5) was calculated by dividing biomass (g) by lactate mass (g) and moles consumed, as described by (White, 2000). Lactate measurements are described below.

Thermodynamic calculations for anaerobic lactate oxidation with nitrate and nitrite were carried out using standard Gibbs free-energy values defined by Thauer *et al.*, (Thauer et al., 1977).

<u>Ion and Gas Chromatography Measurements</u>: New glass IC vials were used for every sample in order to ensure no cross contamination of analytes. Ammonium production via respiratory nitrite ammonification was measured as described by (Yoon et al., 2013). Briefly, because the bacterium simultaneously produces (via dissimilation) and consumes (via assimilation) ammonium, ammonium consumption was first measured with O_2 and lactate by calculating the difference between starting and ending ammonium concentrations. These ammonium consumption values were then normalized to lactate consumed (0.31μmols NH_4^+/lactate) (7.07x10^{-7}μmols NH_4^+/cell calculated from average cell number of stationary phase biomass; Figure S7). Ammonium production during nitrate reducing conditions was then calculated using the mass balance approach from (Giardina and Ryan, 2002) for Total Belowground Carbon Allocation (TBCA) but adapted for nitrogen flux instead of carbon flux:

$$\Delta NH_4^+ = (\Delta lactate_{start\text{-}end} \text{ x } 0.31\mu mols\ NH_4^+/lactate) + \Delta NH_4^+{}_{end\text{-}start} \quad (1)$$

Here, $\Delta lactate_{start\text{-}end}$ (µmols) is multiplied by the ammonium consumed per lactate consumed constant. This value is added to $\Delta NH_4^+{}_{end\text{-}start}$ (µmols), denoted as ending minus starting concentration, which defines whether the change in ammonium is positive (more ammonium produced than consumed) or negative (more ammonium consumed than produced).

Headspace gas from Balch tubes and serum vials was sampled with volume appropriate gastight syringes (Hamilton Company, Reno, NV) pre-flushed with UHP N_2. For high and low nutrient treatments, 10µl and 100µl of headspace were sampled and diluted into 12ml exetainters (Labco, Lampter, Wales, UK) over-pressurized with 15ml UHP N_2, respectively. Similar dilutions were performed for nitrite as e-acceptor experiments, ammonium-free experiments, and time-series experiments. For time-series experiments, an equal volume of headspace gas that was removed at each time-point was replaced with sterile UHP N_2. N_2O and NO were measured by gas chromatography (Shimadzu Greenhouse Gas Analyzer GC-2014) using a 500µl injection volume. The rubber septa on the injection port of the GC was replaced after 100 injections in order to prevent leakage of the sample after the injection needle was lifted out from the injection port. Aqueous concentrations of N_2O were calculated using a Henry's constant of 1.751 (mM (g)/mM (aq)) corrected for the medium's ionic strength and temperature. A total of 8-11 replicates per treatment were analyzed for all experiments discussed in this work (Table S2).

Phylogenetic, Genomic, and Transcriptomic Analysis: A set of 34 NrfA amino acid sequences, representing 33 complete genome sequences and 1 octaheme nitrite reductase (ONR) from known respiratory ammonification organisms were downloaded from GenBank (Table S3). A multiple sequence alignment (MSA) was generated from the sequences annotated as cytochrome c nitrite reductase and ONR using MUSCLE (Edgar, 2004). The resulting alignment was visualized within MEGA5 (Tamura et al., 2011) where the alignment was manually screened for the presence of conserved amino acid residues consistent with those found in NrfA (i.e., heme motifs). A maximum likelihood tree was created from the alignment using RAxML (Stamatakis, 2014) with 500 bootstrap iterations. The presence of NapA, NarG, NirK, and Nor modules were manually queried from each NCBI genome in our set and confirmed by MSA, as described above. Metabolic pathway for pool quinone type was queried on BioCyc Pathway/Genome Database (biocyc.org) for each organism in our set. The structure of *I. calvum*'s NirK protein was predicted using the protein structure predicting algorithm Phyre2 (Kelley et al., 2015). Protein atomic composition for C and N was calculated from amino acid sequences as input files, as described by (Baudouin-Cornu et al., 2004; Grzymski and

Dussaq, 2012), using custom python scripts for each element separately (github.com/dvuono/Cost_minimization).

Due to the high similarity of C5 to 7KIP, reads were aligned to the *Intrasporangium calvum* genomic reference sequence and gtf file (Acc: NC_014830.1) using the STAR RNA-seq aligner (Dobin et al., 2013), with the --limitBAMsortRam parameter set to the recommended value by STAR. Sequence reads were mapped to genomic features to obtain count data using featureCounts (Liao et al., 2014). Systematic changes across experimental conditions were performed on normalized read counts in DESeq2 (Love et al., 2014). The RNA-seq data reported in this study are available within the NCBI BioProject number PRJNA475609.

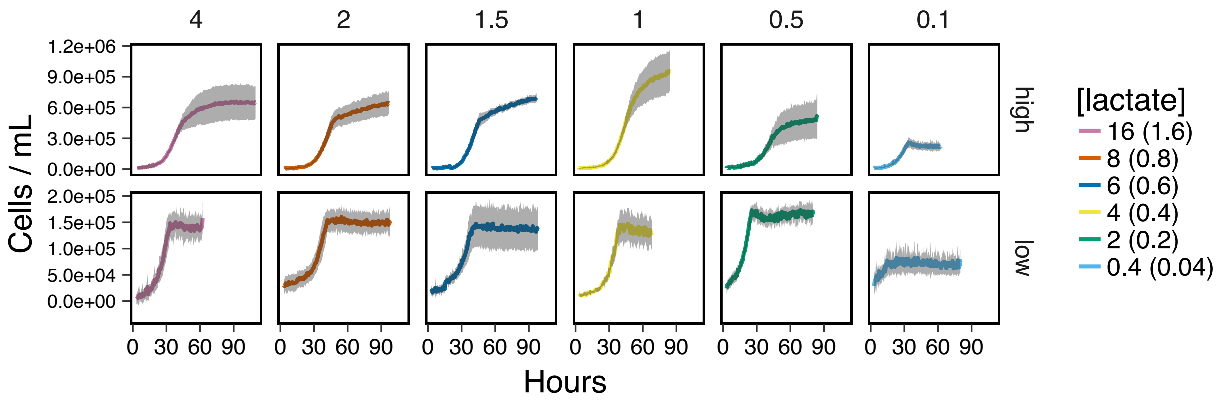

Supplementary Figure 1. Mean cell concentrations for I. calvum cultures grown over a range of C:NO_3^- ratios (columns) at high nutrient (top row) and low nutrient (bottom row) concentrations of the same ratio. Each growth curve consists of n=6 replicates.

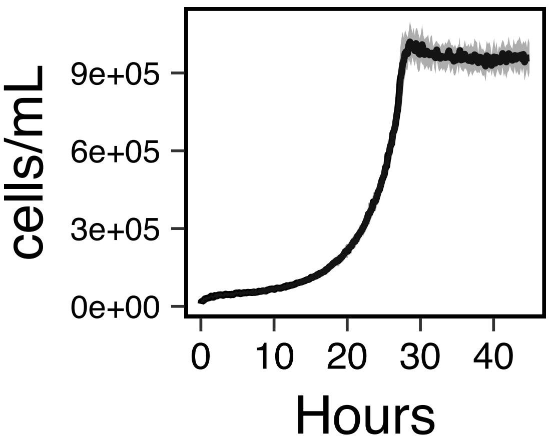

Supplementary Figure 2. Growth curve of I. calvum in a sealed Balch-tube with lactate and O_2 as electron donor/acceptor pair and with ammonium as sole nitrogen source.

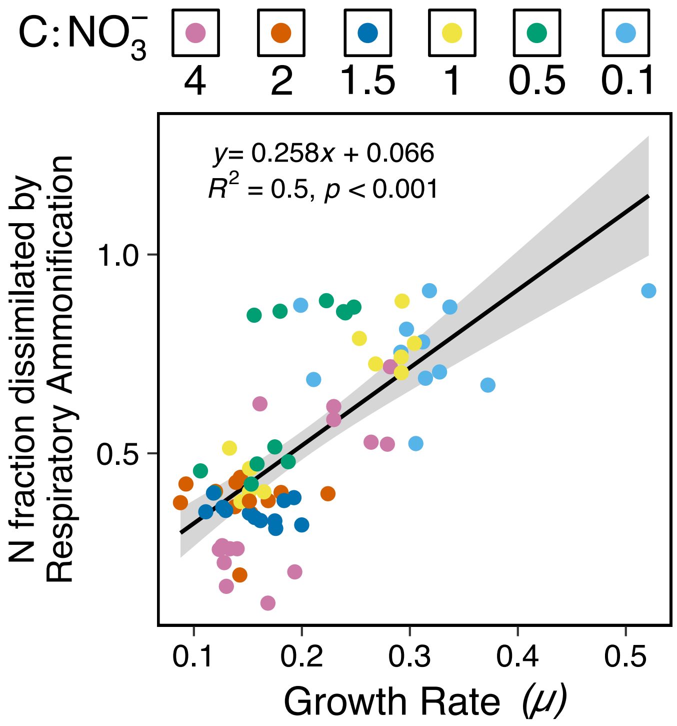

Supplementary Figure 3. Relationship between growth rate and the fraction of N dissimilated by respiratory ammonification for high and low nutrient concentrations. Treatments under C and NO_3^- scarcity, even with low C:NO_3^- ratios, disproportionately produce more ammonium and have higher growth rates.

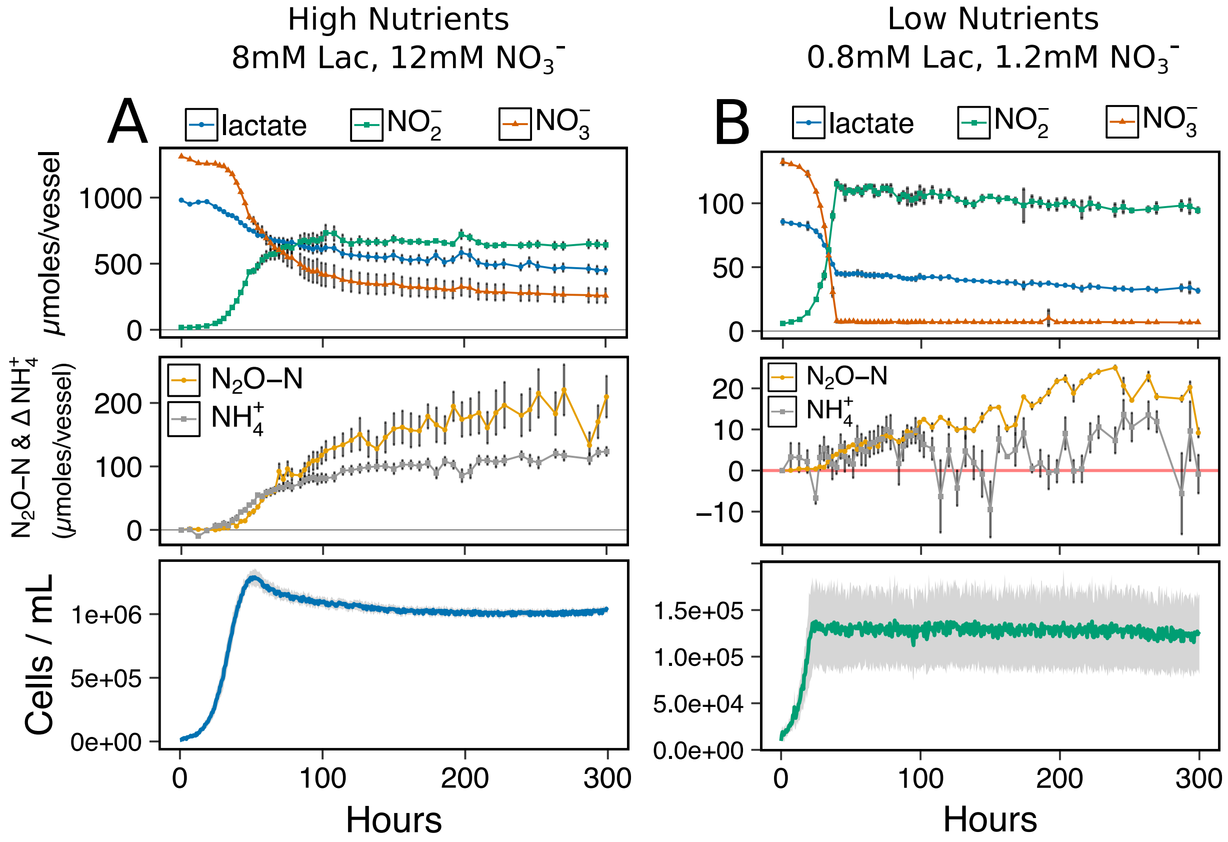

Supplementary Figure 4. Time-series metabolite profiles of a 300-hour incubation for (A) high nutrient and (B) low nutrient concentrations. Shown are the profiles of lactate, nitrate, and nitrite (top pane), production of dissimilated end-products as N_2O-N and net change in NH_4^+ ammonium production (middle pane), and corresponding growth curves of I. calvum cells (C:NO_3^- ratio = 2) (bottom pane).

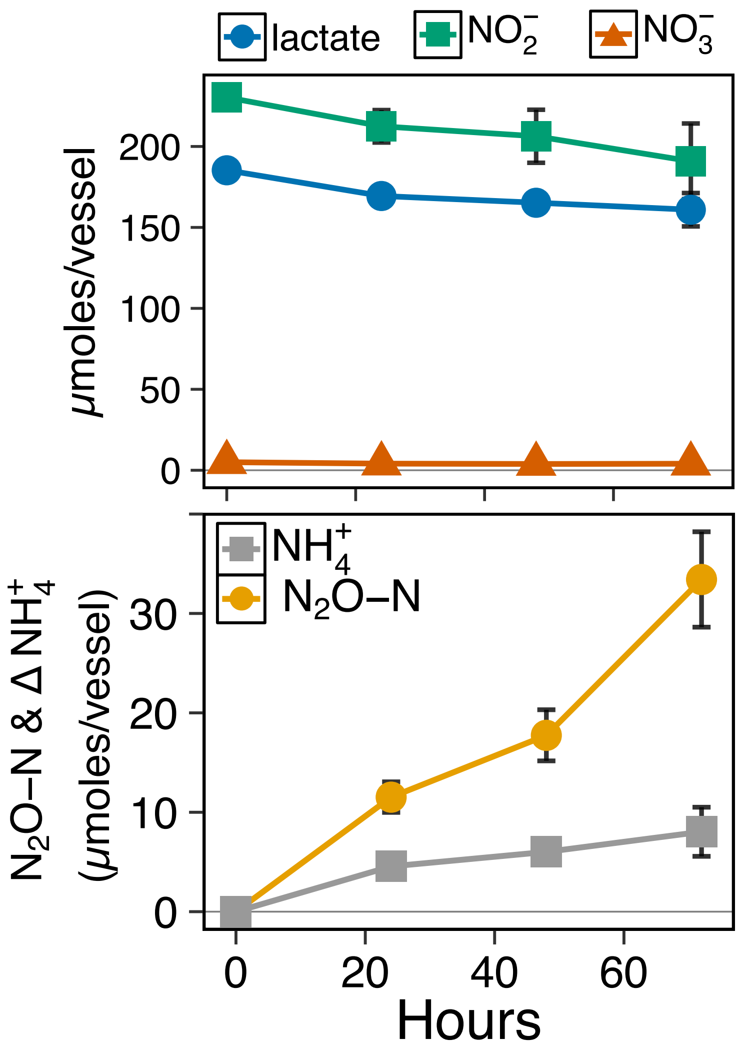

Supplementary Figure 5. Time-series metabolite profiles of a 72-hour incubation conducted in balch-tubes grown under 8mM lactate 12mM nitrite ($C:NO_2^-$ ratio = 2). Profiles for lactate and nitrite (top pane) and production of dissimilated end-products as N_2O-N and net change in NH_4^+ ammonium production (bottom pane).

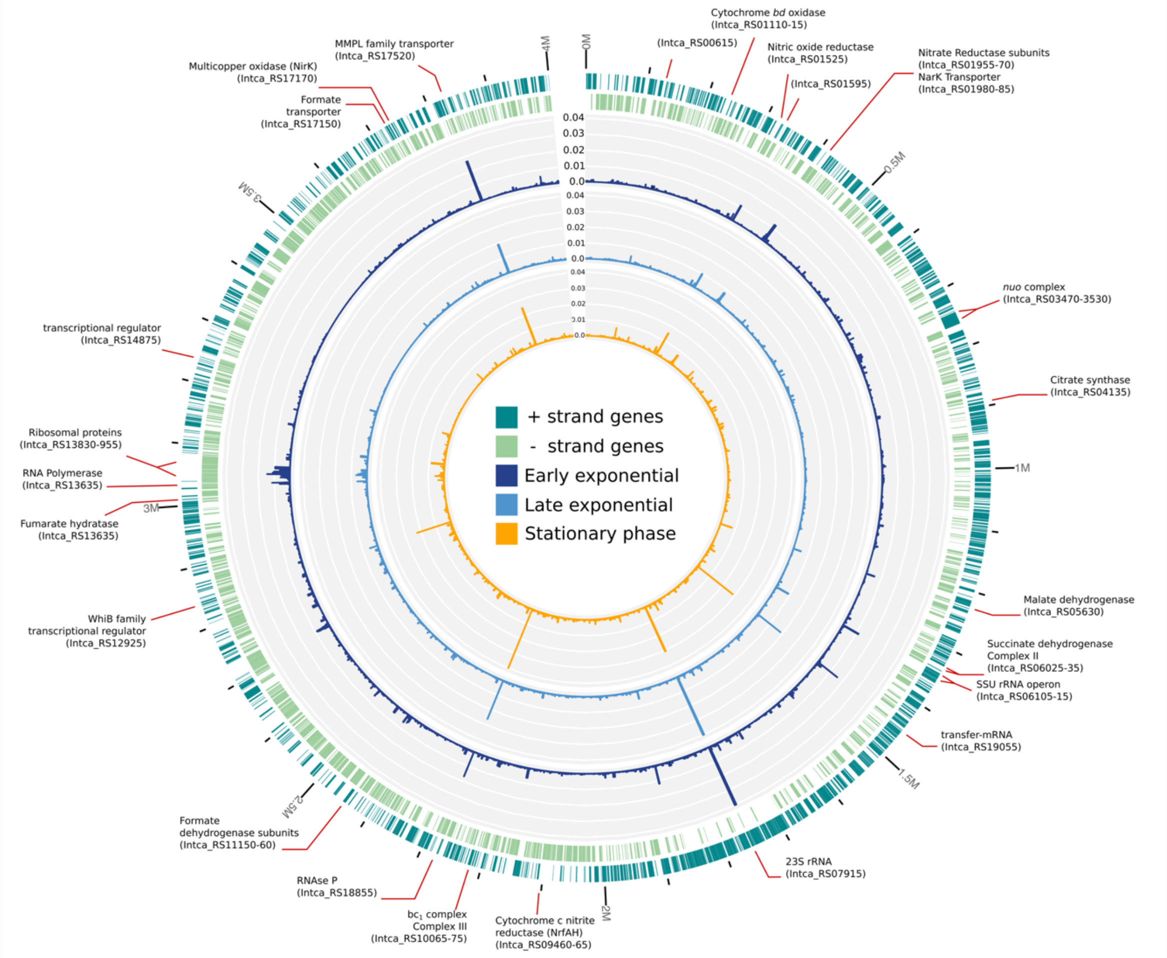

Supplementary Figure 6. The genome-wide transcriptional changes of early exponential, late exponential, and stationary phase I. calvum cells. The first and second outermost rings (dark and light green indicate the open reading frames (ORFs) on the positive and negative strands. The third, fourth, and fifth rings are the relative abundance of transcripts mapped onto the I. calvum genome based on the transcript read counts from early exponential phase, late exponential phase and stationary phase, respectively. The position and locus IDs are marked for the most highly expressed genes and genes involved in the ETC.

Table S1. Literature summary of C:NO_3^- ratio controls on N dissimilation.

Citation	C-source	C:N range	C conc range	NO3 conc range	units	calc method
Kraft *et al.* 2014	amino acids	1.5-3	4.4-43.5	0.5-14.4	mmol	mmol-C/mmol-$\sum NO_x$
Yoon et al. 2015	lactate	1.5-150	0.1-10	0.2	mM	nC*mM-C/nN*mM-N
Van den Berg et al. 2015	acetate	1.8-7.7	160-595	82-93	mg/L	mg-COD/mg-N
Schmidt et al. 2011	Soil organic-C	not specified	2.7-11.4	22.4-79.8	C%,mg-N/kg soil	not specified
Hardison et al. 2015	complex	not specified	C+ - C-	0.6-5	µg	not specified
Fazzolari et al. 1998	glucose	2.5-10	250-1000	100	mg/kg dried soil	mg-C/mg-N
This study	lactate	0.1-4	0.004-16	1.2-12	mM	nC*mM-C/nN*mM-N

Table S2. Summary of all experimental conditions and replicate number in the current study (Figure 2 in main text).

NO_3^- (mM)	Lactate (mM)	Ratio C:NO_3^-	experiment type	ammonium-deplete	replicates	Number of samples taken
1.2	0.04	0.1	end-point	-	9	2
1.2	0.2	0.5	end-point	-	9	2
1.2	0.4	1.0	end-point	-	10	2
1.2	0.6	1.5	end-point	-	10	2
1.2	0.8	2.0	end-point	-	9	2
1.2	1.6	4.0	end-point	-	10	2
12	0.4	0.1	end-point	-	10	2
12	2	0.5	end-point	-	8	2
12	4	1.0	end-point	-	8	2
12	6	1.5	end-point	-	10	2
12	8	2.0	end-point	-	10	2
12	16	4.0	end-point	-	8	2
12	8	2.0	time-series	-	3	17
1.2	0.8	2.0	time-series	-	3	17
12	8	2.0	time-series	-	3	59
1.2	0.8	2.0	time-series	-	3	59
12*	8	2.0	time-series	-	11	4
12	8	2.0	time-series	+	10	3

*nitrite is used as the electron acceptor

Table S3. Organism accession numbers for NrfA and NirK modules.

Organisms	Accession #
	NrfA
Escherichia_coli_K-12	NC_000913.3
Salmonella_enterica CT18	NC_003198.1
Yersinia_kristensenii	NZ_CP009997.1
Yersinia_frederiksenii	NZ_CP009364.1
Vibrio fischeri_ES114	NC_006840.2
S._loihica-PV-4	NC_009092.1
Shewanella_oneidensis_MR-1	NC_004347.2
Desulfotalea_psychrophila_LSv54	NC_006138.1
Sulfurospirillum_deleyianum	NC_013512.1
Wolinella_succinogenes	NC_005090.1
Flexibacter_tractuosus	NC_014759.1
Porphyromonas_gingivalis_W83	NC_010729.1
Symbiobacterium_thermophilum	NC_006177.1
Carboxydothermus_hydrogenoformans	NC_007503.1
Desulfovibrio_vulgaris_Hildenborough	NC_002937.3
Bacillus_vireti	NZ_LDNB01000003.1
Bacillus_bataviensis	NZ_AJLS01000002.1
Bacillus_azotoformans	NZ_AJLR01000001.1
Bacillus_selenitireducens_MLS10	NC_014219.1
Campylobacter_jejuni	NC_002163.1
Opitutus_terrae	NC_010571.1
Anaeromyxobacter_dehalogenans_2_CP-1	NC_011891.1
Rhodopirellula_baltica	NC_005027.1
Intrasporangium calvum 7KIP	NC_014830.1
Intrasporangium calvum C5	This study
Bdellovibrio_bacteriovorus	NC_005363.1
Gimesia_maris	NZ_ABCE01000001.1
Candidatus_Nitrospira_inopinata	NZ_LN885086.1
Myxococcus_xanthus	NC_008095.1
Geobacter_metallireducens_GS_15	NC_007517.1
Geobacter_sulfurreducens_PCA	NC_002939.5
Thioalkalivibrio_nitratireducens	NC_019902.2
Thermodesulfovibrio_yellowstonii_THEYE_A0193	NC_011296.1
	NirK
multicopper_oxidase_[Intrasporangium_calvum]	WP_013494195.1
nitrite_reductase,_copper-containing_[Shewanella_loihica]	WP_011867131.1
nitrite_reductase_[Candidatus_Nitrospira_inopinata]	WP_062488124.1
nitrite_reductase,_copper-containing_[Marivirga_tractuosa]	WP_013454821.1
nitrite_reductase,_copper-containing_[Symbiobacterium_thermophilum]	WP_070105442.1
nitrite_reductase_[Opitutus_terrae]	WP_012373845.1

nitrite_reductase,_copper-containing_[Bdellovibrio_bacteriovorus]	WP_011165004.1
Nitrite_reductase_OS=Bacillus_azotoformans_GN=nirK	ZP_08007035.1
Ochrobactrum_anthropi_ATCC_49188	NC_009668.1
Bradyrhizobium_japonicum_USDA_110	NC_004463.1
Agrobacterium_fabrum_str._C58	NC_003063.2
Sinorhizobium_meliloti_1021	NC_003037.1
Pseudomonas_citronellolis_strain_SJTE-3	NZ_CP015878.1
Rhodanobacter_denitrificans_strain_2APBS1	NC_020541.1
Taylorella_equigenitalis_ATCC_35865	NC_018108.1
Flavobacterium_columnare_ATCC_49512	NC_016510.2
Actinobacillus_suis_ATCC_33415	NZ_CP009159.1
Chromobacterium_violaceum_ATCC_12472	NC_005085.1
Halopiger_xanaduensis_SH-6	NC_015666.1
Halopiger_xanaduensis_SH-6	NC_015666.1
inorhizobium_fredii_HH103	NC_016812.1
Pseudomonas_entomophila_str._L48	NC_008027.1
Pseudomonas_denitrificans_ATCC_13867	NC_020829.1
Flavobacterium_johnsoniae_UW101	NC_009441.1
Rhizobium_etli_CFN_42	NC_007766.1
Ochrobactrum_anthropi_ATCC_49188	NC_009667.1
Caulobacter_segnis_ATCC_21756	NC_014100.1
Rhizobium_giardinii_bv._giardinii_H152	NZ_KB902685.1

Table S4. Concentration and ratio experimental design and produciton values for NH_4^+ and N_2O-N.

[C] (mM)	[NO3] (mM)	C:NO3- ratio	Ammonia per Cell NH4 produced (µmoles)	Ammonia per Lactate NH4 produced (µmoles)	N_2O produced (µmoles)	% Recovery of Dissimilated N
16	12	4	1.94 ± 1.31	7.79 ± 3.3	27.4 ± 7.5	91.64 ± 12.9
8	12	2	4.91 ± 1.07	10.8 ± 4.1	18.1 ± 6.7	72.87 ± 9.3
6	12	1.5	3.07 ± 4.50	10.2 ± 3.8	19.1 ± 6.2	72.77 ± 4.1
4	12	1	8.06 ± 2.19	14.5 ± 4.2	18.1 ± 6.8	61.79 ± 5.1
2	12	0.5	3.82 ± 1.92	8.76 ± 2.9	10.2 ± 3.7	64.21 ± 9.8
0.4	12	0.1	2.05 ± 0.50	1.55 ± 0.2	0.48 ± 0.1	24.44 ± 7.5
1.6	1.2	4	1.12 ± 0.99	2.39 ± 0.7	1.77 ± 0.2	70.47 ± 10.4
0.8	1.2	2	1.50 ± 0.57	2.32 ± 0.5	3.72 ± 0.4	90.71 ± 8.2
0.6	1.2	1.5	0.90 ± 0.53	2.27 ± 0.5	4.53 ± 0.7	88.31 ± 8.6
0.4	1.2	1	1.18 ± 1.17	2.45 ± 0.3	0.88 ± 0.3	50.20 ± 9.4
0.2	1.2	0.5	1.91 ± 0.33	1.13 ± 0.2	0.18 ± 0.0	43.34 ± 20.0
0.04	1.2	0.1	0.03 ± 0.95	0.28 ± 0.2	0.06 ± 0.0	18.10 ± 10.9

Table S5. Growth rate and growth yield values for concentration and ratio experiment.

[C] (mM)	[NO3] (mM)	C:NO3- ratio	Specific Growth Rate (µ)	Doubling Time (hours/generation)	Growth Yield cells (g)/Lac(g)	Molar Growth Yield cells (g)/moles Lac	Growth Yield cells (g)/NO3(g)	Molar Growth Yield cells (g)/moles NO3	Growth Yield cells (g)/NO2(g)	Molar Growth Yield cells (g)/moles NO2
			n=6	n=6	n=3	n=3	n=3	n=3	n=3	n=3
16	12	4	0.143 ± 0.02	4.96 ± 0.73	0.25 ± 0.05	22.3 ± 4.6	0.10 ± 0.02	6.43 ± 1.23	0.63 ± 0.19	28.8 ± 8.6
8	12	2	0.144 ± 0.02	4.86 ± 0.55	0.29 ± 0.02	26.4 ± 1.4	0.12 ± 0.03	7.61 ± 1.77	0.66 ± 0.21	30.1 ± 9.6
6	12	1.5	0.150 ± 0.03	4.77 ± 0.94	n.a.	n.a.	0.11 ± 0.02	6.84 ± 1.45	0.62 ± 0.21	28.6 ± 9.5
4	12	1	0.150 ± 0.01	4.65 ± 0.34	0.26 ± 0.11	23.5 ± 9.6	0.12 ± 0.05	7.46 ± 2.79	0.62 ± 0.25	28.5 ± 11.3
2	12	0.5	0.150 ± 0.03	4.82 ± 1.12	0.27 ± 0.02	24.3 ± 2.2	0.10 ± 0.03	6.38 ± 1.88	0.58 ± 0.18	26.7 ± 8.1
0.4	12	0.1	0.290 ± 0.05	2.46 ± 0.51	0.46 ± 0.003	41.1 ± 0.27	0.15 ± 0.01	9.41 ± 0.66	0.92 ± 0.20	42.5 ± 9.4
1.6	1.2	4	0.241 ± 0.05	2.98 ± 0.69	0.26 ± 0.03	23.5 ± 3.1	0.11 ± 0.01	6.86 ± 0.72	0.83 ± 0.05	38.32 ± 2.48
0.8	1.2	2	0.146 ± 0.05	5.31 ± 1.96	0.29 ± 0.02	25.7 ± 2.2	0.11 ± 0.00	6.99 ± 0.01	0.78 ± 0.06	36.07 ± 2.93
0.6	1.2	1.5	0.164 ± 0.03	4.39 ± 0.97	0.23 ± 0.02	20.8 ± 2.3	0.10 ± 0.00	6.47 ± 0.02	0.63 ± 0.09	29.06 ± 4.20
0.4	1.2	1	0.284 ± 0.02	2.45 ± 0.17	0.27 ± 0.01	23.9 ± 0.5	0.10 ± 0.00	6.51 ± 0.03	0.73 ± 0.09	33.72 ± 4.16
0.2	1.2	0.5	0.214 ± 0.04	3.33 ± 0.67	0.57 ± 0.01	51.4 ± 0.6	0.12 ± 0.00	7.67 ± 0.19	n.a.	n.a.
0.04	1.2	0.1	0.344 ± 0.10	2.16 ± 0.64	n.a.	n.a.	n.a.	n.a.	n.a.	n.a.
